# Tubulin-Destabilizing Agent BPR0L075 Induces Vascular-Disruption in Human Breast Cancer Mammary Fat Pad Xenografts

**DOI:** 10.1371/journal.pone.0043314

**Published:** 2012-08-24

**Authors:** Li Liu, Haley Beck, Xiaolei Wang, Hsing-Pang Hsieh, Ralph P. Mason, Xinli Liu

**Affiliations:** 1 Cancer Imaging Program, Department of Radiology, University of Texas Southwestern Medical Center, Dallas, Texas, United States of America; 2 Institute of Biotechnology and Pharmaceutical Research, National Health Research Institutes, Taipei, Taiwan, Republic of China; 3 School of Pharmacy, Department of Pharmaceutical Sciences, Texas Tech University Health Sciences Center, Amarillo, Texas, United States of America; University of Sheffield, United Kingdom

## Abstract

BPR0L075, 6-methoxy-3-(3′,4′,5′-trimethoxy-benzoyl)-1*H*-indole, is a tubulin-binding agent that inhibits tubulin polymerization by binding to the colchicine-binding site. BPR0L075 has shown antimitotic and antiangiogenic activity *in vitro*. The current study evaluated the vascular-disrupting activity of BPR0L075 in human breast cancer mammary fat pad xenografts using dynamic bioluminescence imaging. A single dose of BPR0L075 (50 mg/kg, intraperitoneally (i.p.)) induced rapid, temporary tumor vascular shutdown (at 2, 4, and 6 hours); evidenced by rapid and reproducible decrease of light emission from luciferase-expressing orthotopic MCF7 and MDA-MB-231 breast tumors after administration of luciferin substrate. A time-dependent reduction of tumor perfusion after BPR0L075 treatment was confirmed by immunohistological staining of the perfusion marker Hoechst 33342 and tumor vasculature marker CD31. The vasculature showed distinct recovery within 24 hours post therapy. A single i.p. injection of 50 mg/kg of BPR0L075 initially produced plasma concentrations in the micromolar range within 6 hours, but subsequent drug distribution and elimination caused BPR0L075 plasma levels to drop rapidly into the nanomolar range within 24 h. Tests with human umbilical vein endothelial (HUVEC) cells and tumor cells in culture showed that BPR0L075 was cytotoxic to both tumor cells and proliferating endothelial cells, and disrupted pre-established vessels *in vitro* and *ex vivo*. In conclusion, BPR0L075 caused rapid, albeit, temporary tumor vascular shutdown and led to reduction of tumor perfusion in orthotopic human breast cancer xenografts, suggesting that this antimitotic agent may be useful as a vascular-disrupting cancer therapy.

## Introduction

Tumor endothelium represents a novel therapeutic target for cancer [1]. Large numbers of neoplastic cells are directly supported by a proportionally small amount of tumor endothelium, making this endothelium critical for both the survival and proliferation of solid tumor mass. Tumor vasculature is distinct from established vascular endothelium in normal tissues in that tumor vasculature is highly permeable (“leaky”), with a relatively high proportion of proliferating endothelial cells and a reduced number of supporting pericytes [2], [3], [4]. Therapeutics that inhibit tumor vasculature development (antiangiogenic therapy) or destroy established tumor vessels (antivascular therapy) exploit differences between tumor and normal vasculature, and have shown promise in preclinical and clinical evaluations [5], [6].

BPR0L075, 6-methoxy-3-(3′,4′,5′-trimethoxy-benzoyl)-1*H*-indole, is a microtubule-destabilizing agent that inhibits tubulin polymerization by binding to the colchicine-binding site [7]. The inhibitory concentration of BPR0L075 that reduces polymerized tubulin by 50% was reported to be 3.3±0.5 µM [7]. Competition binding assays indicated that the binding capacity of BPR0L075 to the colchicine-binding site of tubulin is stronger than that of colchicine (*Ki* values are 0.023 and 1.25 µM, respectively) [7]. BPR0L075 arrests cancer cells at the G_2_-M mitotic checkpoint, and induces cancer cell apoptosis by perturbing mitochondrial membrane potential and activating the caspase-3 cascade [7]. BPR0L075 has shown antiangiogenic activities *in vitro*, evidenced by concentration dependent inhibition of proliferation and migration of endothelial cells and suppression of vascular endothelial growth factor (VEGF)-mediated angiogenesis in Matrigel plug assays [8]. BPR0L075 has also exhibited activity against the growth of human gastric and cervical carcinoma xenografts at doses of 50 mg/kg in nude mice [7], and combination treatments of BPR0L075 and cisplatin demonstrated a synergistic growth inhibition against human lung, colorectal, and cervical tumor xenografts compared to single agent [8].

Agents affecting the cytoskeleton, particularly microtubule-destabilizing agents, such as the vinca alkaloids, colchicines, combretastatins, and ZD6126, have exhibited antivascular effects, producing vascular shutdown and massive tumor necrosis in preclinical and clinical investigations [9], [10]. In the present study, we investigated the vascular disrupting effects of BPR0L075 in orthotopic breast cancer xenografts using a validated dynamic bioluminescence imaging (dBLI) method [11]. We found that BPR0L075 induced rapid, albeit temporary, tumor vascular shutdown and led to reduction of tumor perfusion, suggesting that BPR0L075 can be used as a vascular disrupting agent.

## Results

### *In vivo* imaging of vascular-disrupting effects of BPR0L075

**Figure 1:**
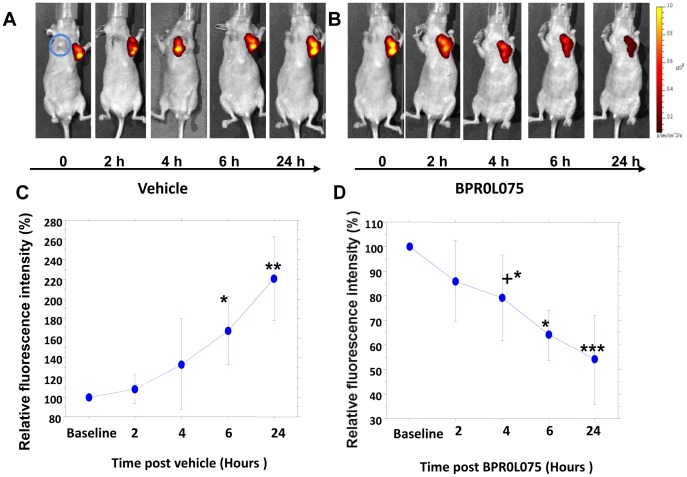
Fluorescence images of mammary breast tumor response to administration of BPR0L075 at successive time points. Sequential images of a single nude mouse with orthotopic MCF7-*lacZ* and MCF7-luc-GFP-mCherry breast tumors growing in the front right (blue circle) and left mammary fat pad, respectively. Fluorescent signal (mCherry) increased over 24 hours following administration of carrier vehicle (A), but signals diminished 4, 6, and 24 hours after injection of BPR0L075 (B); (C) Variation in normalized fluorescent signal intensity for the group of three MCF7-luc-GFP-mCherry tumors in response to vehicle injection; (D) Variation in normalized fluorescent signal intensity for the group of six MCF7-luc-GFP-mCherry tumors in response to injection of BPR0L075. * *P*<0.05, ** *P*<0.01, *** *P*<0.0001; ^+^ only three tumors observed at this time point.

Following implantation of human MCF7-luc-GFP-mCherry cells in the mammary fat pad of nude mice, tumors developed and showed increasing fluorescence as they grew (Figure 1A, C), while contralateral MCF7-*lacZ* tumors showed no fluorescence, as expected (Figure 1A, B). GFP expression was subject to substantial background autofluorescence and is not presented here. The longer wavelength fluorescent protein monomeric (m) Cherry exhibited bright red fluorescence clearly discernible against background and effective for *in vivo* imaging of the transfected tumor tissues. Once tumors reached a volume of about 120 mm^3^, they were evaluated for signal reproducibility and response to BPR0L075. Repeat fluorescent imaging (FLI) of mCherry expression in the tumor over a period of 24 hours showed a steady increase in signal for each of the control mice treated with vehicle attributable to growth, which was significant by 6 hours for the group (n = 3) based on normalized data (*P*<0.05) and at the 24 hour time point based on normalized (*P*<0.005) or absolute photon flux (*P*<0.05) (Figure 1A, C). Following a single i.p. administration of BPR0L075 at 50 mg/kg, there was a significant decline in fluorescent signal within 4–6 hours (n = 6; *P*<0.05) with continued significant decline after 24 hours (*P*<0.001; Figure 1B, D). The FLI study indicates that BPR0L075 has direct toxic effect on tumor cells.

**Figure 2:**
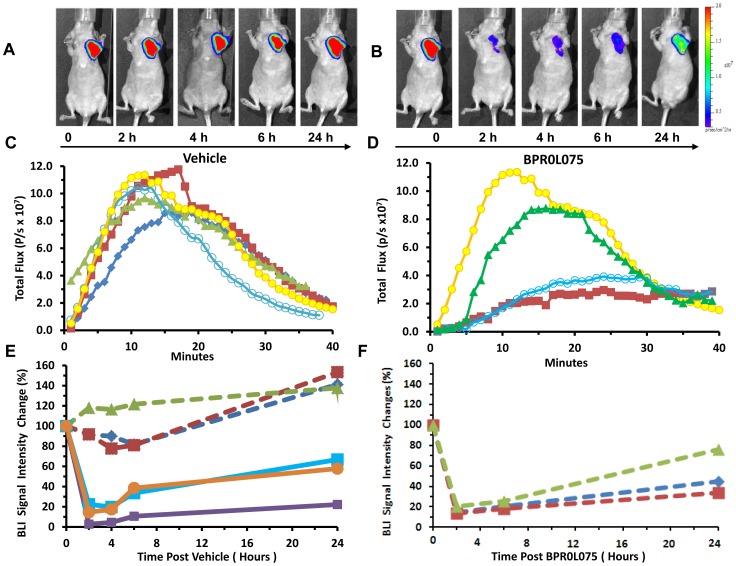
MCF7-luc-GFP-mCherry breast tumor response to administration of BPR0L075 observed by bioluminescence. (A, B) BLI of the same mouse as shown in Figure 1 with images obtained within 15 mins after administration of luciferin s.c. immediately after the FLI; (C) Dynamic BLI showing evolution of signal intensity for the MCF7-luc-GFP-mCherry tumor shown in “A” over 40 minutes; respective curves show baseline (blue diamonds), together with 2 (red squares), 4 (green triangles), 6 (open circles) and 24 (yellow circles) hours after administration of drug carrier vehicle; (D) Dynamic BLI with respect to treatment with BPR0L075 (50 mg/kg i.p.): baseline (same as 24 hour time point in “A”, yellow circles); 2 hours after drug (red squares), 6 hours after drug (open circles) and 24 hours after drug (green triangles); (E) Normalized maximum bioluminescent signal observed in the first 15 mins post luciferin injection at each time point sequentially for individual mice over 24 hours with respect to carrier vehicle (n = 3, dashed lines) or drug (n = 3; solid lines). Control mice show highly reproducible signal, though with significant increase after 24 hours, whereas treated mice show severely decreased signal at the 2 hour time point, though with progressive recovery over 24 hours; (F) The control mice in “E” (dashed lines same symbol designation) were treated with drug on the second day and maximum light emission is plotted. These data were renormalized to the 24 hour time point in “E”. Data look very similar to the mice receiving drug in “E”.

Administration of *D*-luciferin substrate generated bioluminescent signal in the MCF7-luc-GFP-mCherry tumors (Figure 2A), which increased rapidly typically reaching a maximum within 10 to 15 mins followed by decline over the next 30 mins (Figure 2C). The kinetics of light emission was highly reproducible for each mouse following administration of vehicle (DMSO/Tween/saline) and fresh luciferin at 2, 4, 6, and 24 hours (Figure 2C). Maximum signals were recorded and normalized signals were found to be quite stable for the group of control mice with no significant change until the 24 hour time point (n = 3, *P*>0.05). Following administration of BPR0L075 (Figure 2B), significant decrease in normalized signal was observed within 2 hours (n = 6, *P*<0.0001; Figure 2B, D, E, F), which remained significantly depressed for 24 hours (*P*<0.001), although the 24 hour time point showed significant recovery compared with the 2, 4, and 6 hour time points (*P*<0.001). Each individual tumor showed highly consistent response with at least 80% reduction in emitted signal intensity at 2 hours (Figure 2E, F). Similar behavior was seen in response to drug, whether or not tumors had been part of the initial control (vehicle only) study. We note that bioluminescent signal intensity was still declining 40 minutes after injection of substrate (Figure 2C, D), and thus, we tested for residual light emission in select mice at the 2 hour time point prior to administration of additional fresh luciferin. Following vehicle, the residual signal was <2×10^6^ photons/s, as compared with 9×10^7^ photons/s following additional luciferin. At 2 hours following BPR0L075 residual signal was <5×10^6^ photons/s, as compared with >1.6×10^7^ photons/s following additional luciferin confirming minimal interference in sequential measurements.

Similar dBLI results were observed using MDA-MB-231-luc mammary fat pad xenografts. Administration of *D*-luciferin substrate to vehicle-treated control mice generated highly reproducible bioluminescent signals, which peaked after 12 mins and then declined (Figure S1A, B). No significant differences were observed for the light emission curves at 2, 4, and 24 hours post vehicle injection following administration of fresh luciferin on each occasion (*P*>0.05; Figure S1B). Meanwhile, light emission was significantly lower 2 and 4 hours after BPR0L075 treatment, with only 11.7±7.8% and 26.9±13.1% of initial light emission intensity, respectively (*P*<0.01; Figures S1C, D, & S2). Overall normalized maximum BLI signal intensity at 24 hours recovered to 55% of pre-therapy level, although one mouse showed persistent suppression of BLI signal (Figure S2).

### Histological validation of decreased tumor perfusion after BPR0L075 treatment

**Figure 3:**
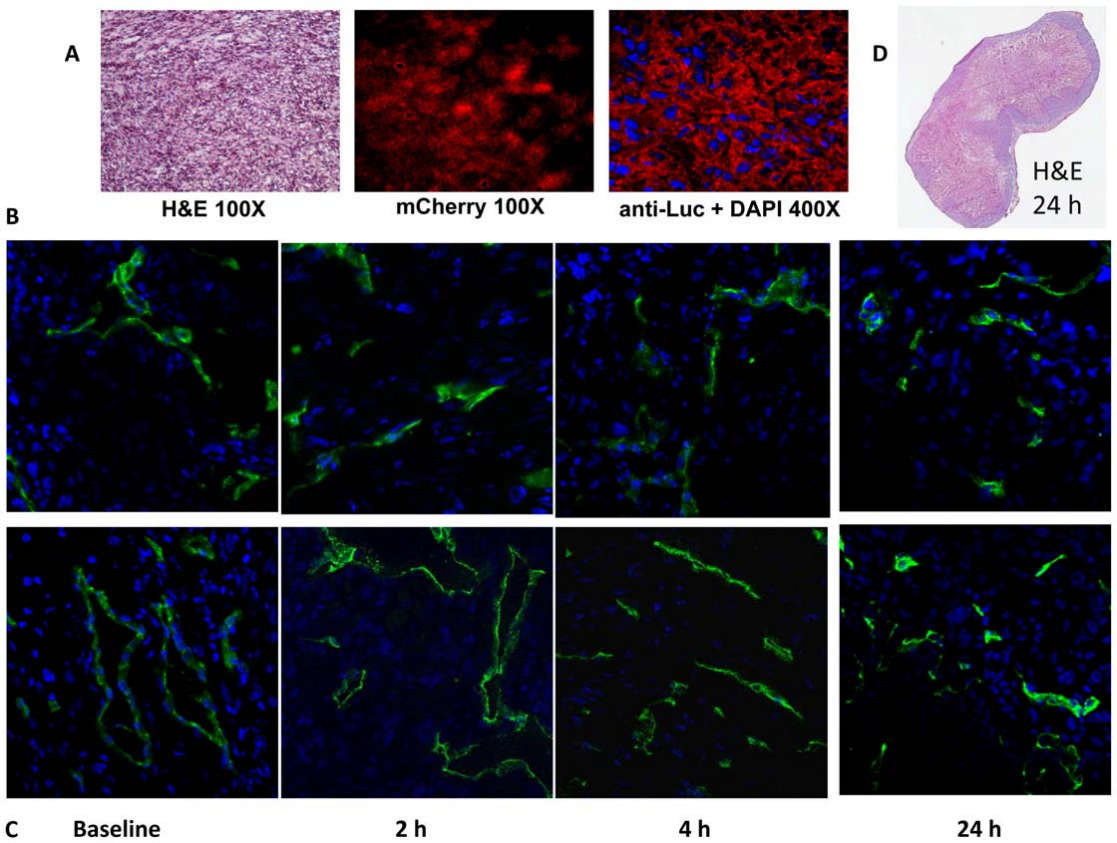
Histological validation of temporary vascular shutdown in MCF7-luc-GFP-mCherry mammary fat pad tumors with respect to BPR0L075 administration. (A) Tumor tissues from control mouse 4 hours after vehicle treatment showing H&E staining (original magnification ×100), mCherry fluorescence signals (×100), and luciferase expression stained with anti-luciferase antibody (red) with DAPI (blue) (magnification ×400); (B) Tumor sections from four control tumors showing vascular extent based on anti-CD31 (green) and perfusion marker Hoechst 33342 (blue); and (C) corresponding tumor sections from four tumors in mice treated with BPR0L075 (50 mg/kg i.p.) at different time points (magnification ×400). (D) Whole mount H&E section 24 hrs after administering BPR0L075 showing about 70% necrotic fraction.

At various time points mice were injected with the fluorescent dye Hoechst 33342 before euthanasia and tumors were excised for histological examination. For MCF7-luc-GFP-mCherry tumors, H&E staining revealed the typical histological appearance of tumors and the mCherry and luciferase expression were confirmed by fluorescence microscopy (Figure 3A). Tumor vascular density and perfusion were assessed based on anti-CD31 staining (green, tumor vasculature marker) and distribution of Hoechst 33342 (blue, perfusion marker) (Figure 3B, C). The control tumors showed extensive well perfused vasculature at baseline and following administration of vehicle, without any significant changes in the fluorescence intensity of CD31 staining and Hoechst distribution (*P*>0.05) (Figure 3B). In BPR0L075 treated tumors (Figure 3C), blood vessels were found to be distributed throughout the tumor based on anti-CD31 staining at each time point with no significant change (*P*>0.05), but the fluorescence intensity and distribution of Hoechst perfusion marker were significantly lower, particularly at 2 and 4 hours, with only 23±4% and 46±3% of baseline values (*P*<0.01, Figure 3C), though with recovery at the 24 hour time point (80±10%; *P*>0.05). The H&E stained sections showed that control MCF7-luc-GFP-mCherry tumors exhibited substantial necrosis with a wide range of necrotic fractions (28.6±14.9 SE%) and this was greater following treatment with BPR0L075 (62.6±7.4 SE%, Figure 3D), though did not reach significance (*P*>0.05). Histology of the MDA-MB-231-luc tumors confirmed similar behavior in the control (vehicle; Figure S3A) and treated (BPR0L075; Figure S3B) tumors. Quantitative analysis of Hoechst 33342 staining in tumor sections revealed the BPR0L075 treatment significantly decreased the intensity to 49±5% and 72±15% of the baseline values at 2 and 4 hour, respectively (*P*<0.01, Figure S3B). The tumors perfusion recovered to 78±6% at 24 hour post drug therapy (Figure S3B). The microvessel density (CD31 staining) was also significantly decreased to 56±2% (2 hours) and 62±9% (4 hours) of baseline values in BPR0L075 treated MDA-MB-231-luc tumors (*P*<0.01, Figure S3B). Necrosis assessed from whole mount tumor sections was significantly increased in treated tumors 24 hrs after BPR0L075 administration compared with vehicle controls (45.1±13.9 SE % vs. 14.5±8.0 SE%; *P*<0.05, Figure S3).

### Pharmacokinetics of BPR0L075 treatment

**Figure 4:**
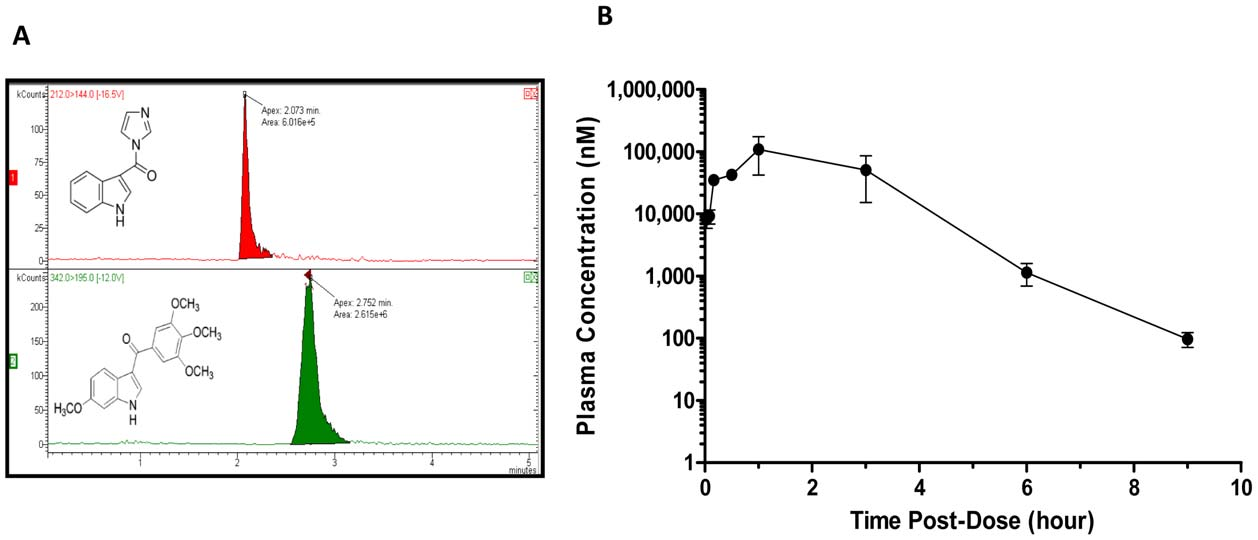
Plasma pharmacokinetics of BPR0L075 in CD-1 mice after administration of drug at 50 mg/kg intraperitoneally. (A) LC-MS/MS analysis of BPR0L075 with chromatogram showing the retention time of internal standard [1-(1H-indol-3-ylcarbonyl)-1H-imidazole] and BPR0L075 as 2.1 and 2.8 min, respectively. (B) The plasma concentration-time curve following BPR0L075 treatment.

To correlate the acute vascular-disrupting activity of BPR0L075 to drug levels *in vivo*, the plasma pharmacokinetics of BPR0L075 was assessed in normal mice using a LC-MS/MS method. The LC chromatograms are shown in Figure 4A. The concentration-time profile is shown in Figure 4B. After a single dose of BPR0L075 (50 mg/kg, i.p.), the concentration peaked at 108±66 µM at 1 hour, and declined to 1 µM after 6 hours, with around 0.1 µM after 9 hours. The drug level was below the limit of quantification at 24 hour post therapy.

### BPR0L075 inhibits proliferation of endothelial cells

**Figure 5:**
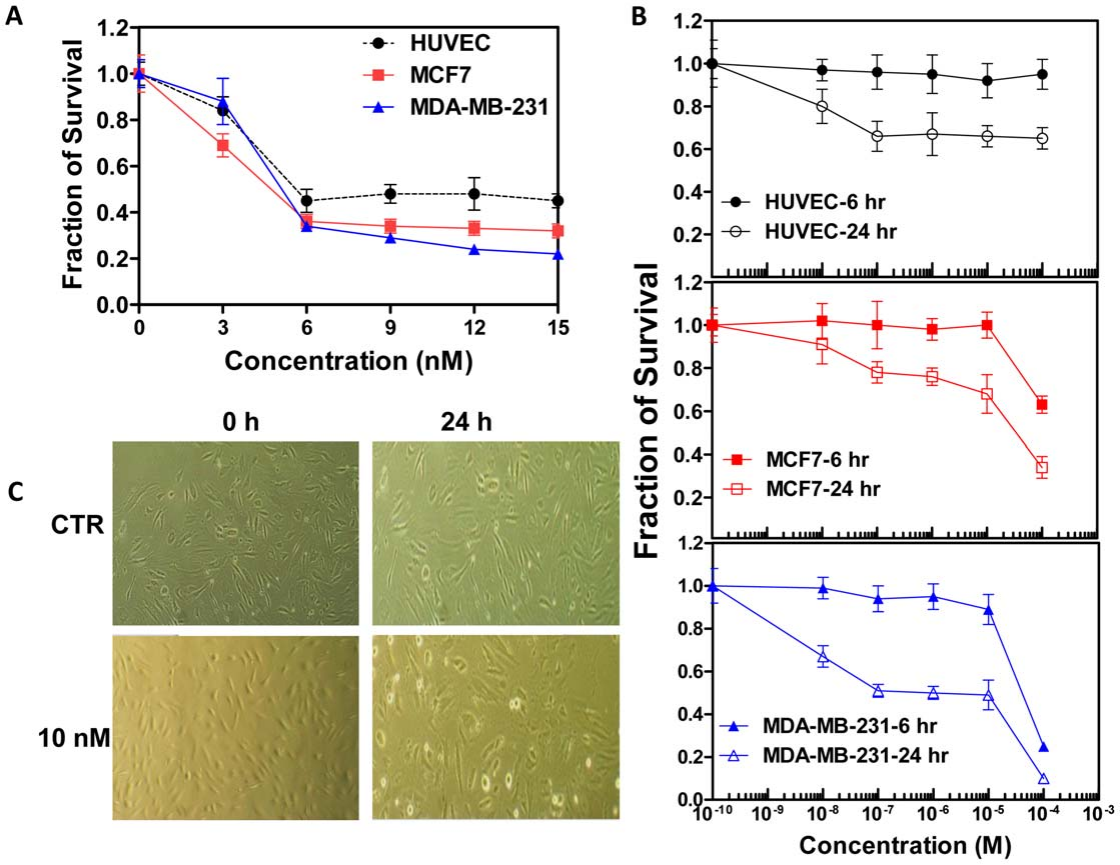
Inhibition of cell proliferation by BPR0L075. (A) Proliferating human umbilical vein endothelial cells (HUVEC), and breast cancer (MCF7 and MDA-MB-231) cells were treated with BPR0L075 at various concentrations (0–15 nM) for 96 hours and the viable cells were stained with sulforhodamine B. (B) HUVEC and tumor cells were treated with BPR0L075 at various concentrations for 6 hours and 24 hours and cytotoxicity was evaluated by SRB assay. Fraction of cell survival relative to controls represents mean ± SD of 16 determinations. (C) Representative photomicrographs show the morphology of HUVEC cells in the presence of ethanol vehicle or 10 nM BPR0L075 in medium. All experiments were repeated three times.

BPR0L075 displayed cytotoxic effects on both MCF7 and MDA-MB-231 tumor cells, as well as proliferating HUVEC cells in the dose range of 3–15 nM after a 96-hour exposure, with an IC_50_ value of 5.5 nM for tumor cells and 6.0 nM for proliferating HUVEC cells (Figure 5A). The BPR0L075 induced cytotoxicity in HUVEC cells was concentration and time dependent (Figure 5A, B). In the 10 nM-100 µM dose range, BPR0L075 was not cytotoxic to the HUVEC cells after short incubation time (6 hours), but was toxic after 24 hours exposure (Figure 5B). BPR0L075 treatment also induced concentration- and time-dependent cytotoxicity in MCF7 and MDA-MB-231 tumor cells (Figure 5B). BPR0L075 treatment (10 nM for 24 hours) changed the morphology of proliferating endothelial cells, as the cells retracted and rounded up, formed intercellular gaps and lost their cell-cell contact, while the control endothelial cells remained flat (Figure 5C).

### BPR0L075 disrupts pre-established HUVEC networks

**Figure 6:**
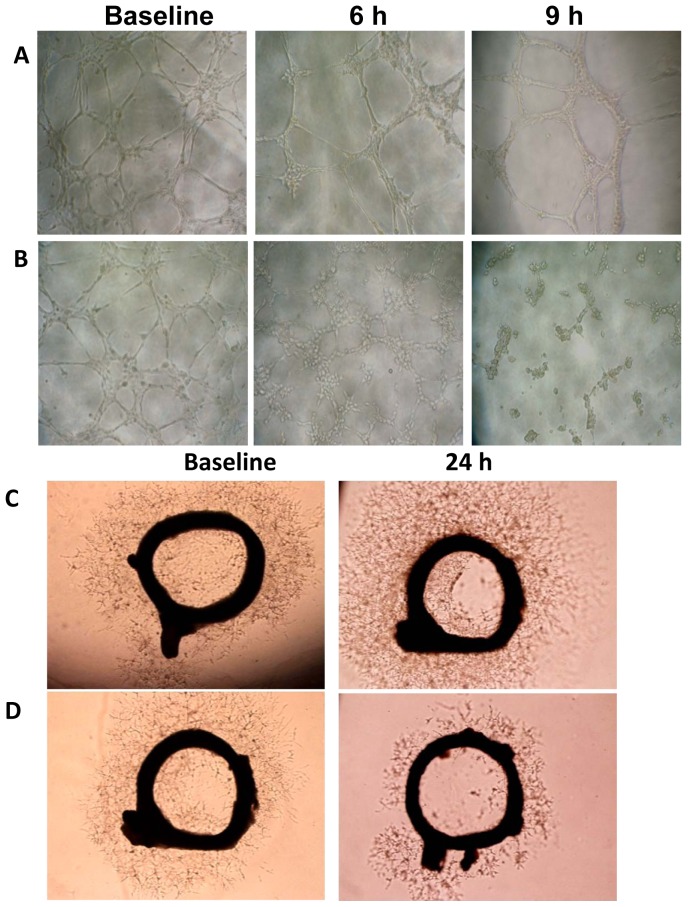
BPR0L075 disrupts pre-established HUVEC capillary-like networks. HUVEC cells were seeded in a 96-well tissue culture plate coated with Matrigel for 12 hours before exposure to ethanol vehicle (A) or 10 nM of BPR0L075 (B) and cells were photographed at different time points. BPR0L075 decreased vessel sprouting *ex vivo*. Incubation of freshly isolated rat aorta (1 mm thick rings) in 1∶1 Matrigel and RPMI medium containing 20 ng/mL VEGF generated vessel sprouts after 48 hour (baseline). Medium was replaced with fresh medium containing ethanol vehicle (C) or 10 nM of BPR0L075 (D) for an additional 24 hour. Representative images show massive vessel spouting in control rings, but reduced vessel sprouting in BPR0L075 treated rings. All experiments were repeated three times.

Endothelial cells plated on Matrigel in the presence of VEGF aligned and formed capillary tubular networks (baseline, Figure 6A, B). Incubation of endothelial cells in the presence of ethanol vehicle did not affect the cellular capillary network (Figure 6A). However, after a 6-hour treatment with BPR0L075 (10 nM, a nontoxic condition, Figure 5B), the pre-established vessels were disengaged, and endothelial cells dropped out of the capillary networks by 9 hours (Figure 6B). We confirmed the vascular disrupting effects of BPR0L075 on newly established blood vessel sprouts (neovessels) growing from aortic ring explants (Figure 6C, D). VEGF (20 ng/mL) stimulated microvessel sprouting leading to outgrowths of networks of vessels around the rat aortic rings (baseline, Figure 6C, D), while ethanol vehicle did not affect the microvessel sprouting (Figure 6C), treatment with BPR0L075 (10 nM for 24 hours) resulted in a reduction of vessel sprouting (Figure 6D).

## Discussion

Tumor vasculature is an attractive therapeutic target, since differences in the structure and physiology of immature tumor versus mature normal vasculature provide an opportunity for the selective disruption of tumor endothelium, and ultimate destruction of tumor cells that depend on functional blood vessels [1], [3]. Tumor blood vessels consist of a chaotic network of tortuous, thin-walled vessels with a significant degree of neovasculature and a relatively high proportion of proliferating endothelial cells [4], [12]. Most of the vascular endothelium in a normal adult is thought to be quiescent, with only 0.01% of endothelial cells undergoing division; however, in tumors, the expression of proangiogenic growth factors can result in an endothelial cell proliferation rate 35-fold higher than that of normal tissues [13], [14]. Additionally, tumor vessels are deficient in pericyte coverage, becoming leaky, fragile, susceptible to disruption, and prone to spontaneous hemorrhaging and thrombosis [1], [15]. Targeting the tumor vasculature presents unique opportunities for therapeutic intervention in cancer treatment. Two distinct classes of drugs have been used for vascular targeting: antiangiogenic agents that inhibit the formation of new vessels, and vascular disrupting agents (VDAs) that selectively damage established tumor vasculature [6], [16], [17].

BPR0L075 is a heterocyclic 3-aroylindole tubulin-binding compound, structurally related to the classical tubulin-binding and vascular disrupting agents, colchicines, and combretastatin A4 phosphate (CA4P). Previous studies have shown that BPR0L075 interferes with tumor cell microtubule dynamics, which leads to cancer cell cycle arrest and cell death by apoptosis [7] and antiangiogenic effects [8]. The current study shows that in addition to the direct cytotoxic effect on tumor cells, BPR0L075 also disrupts tumor vasculature and shuts down tumor blood perfusion *in vivo*.

We showed that acute administration of a single dose of BPR0L075 (50 mg/kg) caused rapid, extensive vascular shutdown in both human mammary MCF7-luc-GFP-mCherry and MDA-MB-231-luc orthotopic breast tumors based on dynamic BLI (Figure 2, S1, S2) and confirmed by immunohistochemistry (Figure 3, S3). The extent of vascular shutdown was quite similar (about 80% assessed by dBLI) at 2 hours despite significantly different tumor growth rates. There was no change in the vascular extent during the acute period as judged by CD31 expression (MCF7-luc-GFP-mCherry tumor), however distribution of Hoechst dye clearly confirms significantly reduced perfusion. At earliest times Hoechst dye is confined to the vascular compartment itself, whereas here it appears to have extravasated and labeled cell nuclei. Clearly, this can only occur in regions of tumor which have perfused vasculature.

We recently demonstrated the utility of firefly luciferase-based dynamic BLI to assess drug-induced acute vascular changes [10], [11] and others confirmed that dynamic evolution of BLI signal could be related to angiogenesis, validated using Evan's blue dye [18]. Repeat BLI showed highly reproducible dynamic evolution of signal intensity following subcutaneous injection of luciferin substrate in the neck region of mice with maximum signal detectable after 10 to 15 minutes. For the control mice BLI signal was stable within 5% for repeat measurements over 6 hours, but showed significant increase on the following day, which we attribute to tumor growth. Following BPR0L075 treatment, significantly less signal was generated following fresh luciferin administration after 2 hours (>80% reduction) and signal generation remained depressed 24 hours later, though with significant recovery compared with the early acute time points. We attribute these changes in emitted light to vascular disruption and diminished delivery of luciferin substrate to the luciferase expressing breast tumor cells. Indeed, we have previously observed similar behavior in MDA-MB-231-luc breast tumor cells growing as xenografts in mice with respect to a single dose of CA4P (120 mg/kg) [11], a VDA currently under phase III clinical evaluations. Those results were validated by comparative dynamic contrast enhanced MRI [11]. In both the current study and previously with CA4P, we used immunohistochemistry to verify that vascular perfusion was significantly reduced (Figure 3, S3). In both cases, signal recovery indicative of vascular perfusion was seen 24 hours later. Given the residual BLI signal from previous measurements sometimes seen at the start of successive time courses, the extent of vascular shutdown was actually a little greater than estimated.

It was previously reported that repeat doses of BPR0L075 caused tumor growth delay even in multi-drug resistant tumors such as P-gp170/MDR-overexpressing KB-VIN10 human cervical carcinoma [7]. Ultimate clinical application will require coordination with additional drugs or irradiation, and thus, insight into vascular shutdown will be crucial for timing considerations. Vascular shutdown would be expected to cause hypoxiation [19], [20] with consequent radioresistance, and influence delivery of other chemotherapeutic drugs, as reviewed extensively [21]. Indeed, multiple imaging techniques can provide insight into tumor vasculature non-invasively, as reviewed recently [10], [22]. Dynamic BLI has the distinct advantage of providing facile economical high throughput evidence of vascular perturbation, though of course the approach does require luciferase-transfected cells. A bonus is that luciferase expressing cells also report on tumor growth during a long-term study [18], [23]. In addition, for MCF7-luc-GFP-mCherry mammary fat pad xenografts, fluorescence imaging confirmed rapid tumor growth over 24 hours and decreased FLI signal after BPR0L075 treatment suggested cell death following BPR0L075 administration (Figure 1), although hemorrhage may also contribute to light absorption. We note that BLI signal response to BPR0L075 was much more rapid than FLI (compare Figures 1 and 2). We believe this represents the rapid vascular shutdown and hence lack of delivery of fresh luciferin substrate. Meanwhile, mCherry fluorescent protein is reported to have a half-life of about 24 hours [24]. Thus, if tumor cells were killed then no new synthesis would occur, but about 50% of the protein would remain after 24 hrs, as was observed in Figure 1B and D. The BLI signal decreased faster and further, but also recovered by 24 hrs. We believe this reflects vascular access of a sub-saturating luciferin dose. Indeed, it was reported that higher doses of luciferin can yield much greater light emission [23].

The pharmacokinetic study showed that BPR0L075 cleared rapidly from the body with C_max_ = 108±66 µM and T_max_ = 1 hour after an intraperitoneal dose at 50 mg/kg, with the levels around 0.1 µM after 9 hours (Figure 4B). The pharmacokinetic time course of BPR0L075 coincided well with the timing of BPR0L075 induced vascular shutdown effects *in vivo*. Approximately 80% reduction of tumor perfusion were observed 2 hours after therapy, persisting at 4 and 6 hours, then gradually recovering by 24 hours (Figure 2, S1), a time point when the plasma drug level dropped to low nanomolar range (Figure 4B). The results indicate that the BPR0L075 induced rapid and temporary vascular shutdown effect is time dependent.

BPR0L075 was cytotoxic to proliferating endothelial cells *in vitro* after prolonged exposure (Figure 5A); it altered HUVEC cell morphology with cellular retraction and increased formation of intercellular gaps due to loss of cell-cell contact (Figure 5C). We hypothesize that the change in HUVEC cell morphology led to vascular disruption, since BPR0L075-induced vascular endothelial cell death was too slow to account for the rapid vascular shutdown effects observed in animal models, where maximal effects on tumor blood flow occurred between 2 and 4 hours in the two orthotopic breast cancer models (Figure 2, S1, S2). Meanwhile, HUVEC cell death was observed after 24 to 96 hours of continuous exposure of BPR0L075, with no effect at 6 hours (Figure 5A, B).

BPR0L075 treatment also disrupted pre-established vasculature *in vitro* and *ex vivo* (Figure 6). Previous reports have shown antiangiogenic effects of BPR0L075, in which the drug was added at the beginning of Matrigel plug and aortic ring assays, indicating that BPR0L075 blocks formation of vascular structures or has antiangiogenic effects [8]. In the current study, BPR0L075 treatment caused collapse of pre-established vasculature *in vitro* and *ex vivo*, which may potentially contribute to the loss of tumor blood vessel integrity *in vivo*, leading to compromised delivery of substances to tumor, including the luciferin substrate. Tumor vessels are known to be susceptible to microtubule-binding agents [9], [10], [25], [26], [27]. Highly proliferative endothelial cells in the immature tumor vasculature are considered to increasingly rely on microtubule network to maintain their elongated, three-dimensional shape, thus rendering them intrinsically sensitive to disruption by agents that bind to tubulin and affect the endothelial cells' cytoskeleton [28], [29], [30], [31]. Our data indicate that microtubule-destabilizing agent BPR0L075 is an effective vascular disrupting agent.

In addition to its vascular disrupting activity *in vitro* and *in vivo*, BPR0L075 also has direct cytotoxicity in MCF7 and MDA-MB-231 breast cancer cells (Figure 5A, B). Previous study has shown that BPR0L075 exhibited broad cytotoxicity in a variety of human tumor cell lines by arresting the cells in G_2_-M phase and inducting apoptosis [7]. After 24 hrs increased necrosis was observed in both tumor types, but it only reached significance for the MDA-MB-231-luc tumor sections (Figure S3). The FLI and BLI study of MCF7-luc-GFP-mCherry mammary fat pad xenografts indicated that BPR0L075 not only has direct toxic effects on rapidly proliferating tumors (Figure 1), but also affects the tumor microenvironment by decreasing tumor perfusion (Figure 2, 3, S1, S2, S3). The previous reported antitumor activity of BPR0L075 in human cervical carcinoma xenografts [7] was likely a result of a combination of direct cytotoxic activity against the tumor cells and tumor vascular disruption effects. Our study indicates that BPR0L075 is a dual activity agent capable of generating both a vascular targeting effect and direct tumor cell cytotoxicity.

## Conclusions

In summary, BPR0L075 treatment caused rapid, though temporary, tumor vascular shutdown and led to reduction of tumor perfusion in human breast mammary fat pad xenografts, likely due to direct vascular disrupting effect on the endothelial cells. These observations indicate that BPR0L075 possesses vascular-disrupting activity in addition to antimitotic activity. This knowledge can help guide rational selection of appropriate doses and administration schedules to maximize the therapeutic potential of BPR0L075.

## Materials and Methods

### Chemicals

BPR0L075 (molecular weight: 342 Da) was synthesized as described previously [32]. It is a white solid, which was dissolved in ethanol as a 2 mM stock solution for *in vitro* evaluation. For use *in vivo*, BPR0L075 was dissolved in DMSO/Tween-80 (1∶3, vol/vol) cosolvent, and then diluted three fold with saline, sterile filtered, and used immediately for injection (dosing solution concentration of 8.33 mg/mL). Matrigel was obtained from BD Biosciences (San Jose, CA). All other chemicals were purchased from Sigma-Aldrich (St. Louis, MO), and were of standard analytic grade or higher.

### Cell culture

Human umbilical vein endothelial cells HUVEC (ATCC CRL-1730) were purchased from American Type Culture Collection (ATCC, Manassas, VA) and used between the second and third passages. Cells were maintained in ATCC F-12K medium, supplemented with 10% fetal bovine serum (FBS; Invitrogen, Carlsbad, CA), 0.1 mg/mL heparin (18 U/mL, from porcine intestinal mucosa; Sigma), and 27 µg/mL endothelial cell growth supplement (ECGS, from bovine pituitary; Sigma). Cells were cultured in tissue culture flasks coated with 0.1% gelatin solution and grown in an incubator with 95% air and 5% CO_2_. Human breast cancer cells MCF7 and MDA-MB-231 (ATCC, Manassas, VA) were infected with a lentivirus containing firefly luciferase (luc) reporter gene, and a highly expressing reporter clone was isolated. Cells were also stably transfected with the GFP and mCherry or *lacZ* gene as described previously [33]. All breast cancer cells were cultured in RPMI-1640 medium (Mediatech, Inc., Manassas, VA) with 10% FBS, 100 U/mL penicillin, and 100 µg/mL streptomycin and grown in an incubator with 95% air and 5% CO_2_.

### Animals

Athymic female nude mice (NCI, Frederick, MD), aged 6–8 weeks were used for imaging studies. Female *CD*-*1 mice* [Crl: CD1 (ICR), Charles River Laboratories], weighing 25–35 g, were used for pharmacokinetics study. All animal procedures were performed according to the Institutional Animal Care and Use Committee approved protocols (Texas Tech University Health Sciences Center protocol #09001 and UT Southwestern Medical Center APN #2009-0150 and 2008-0270).

### *In vivo* optical imaging

MCF7-*lacZ* and MCF7-luc-GFP-mCherry cells (1 million each) were implanted into the right and left mammary fat pads of six nude mice, respectively, and tumors were allowed to grow to about 5 mm in diameter prior to imaging. A subset of these tumors served as the control group (n = 3). Tumor volume was measured by caliper and calculated with the formula V = 0.5×length×width^2^. Bioluminescent and fluorescent imaging were performed using a Caliper Xenogen IVIS® Spectrum (Caliper Life Sciences, Hopkinton, MA). Mice were anesthetized with 1% isoflurane (Isothesia®, Butler, Dublin, OH) in oxygen. A light image was obtained for anatomical orientation and fluorescent imaging performed to observe mCherry using standard parameters including 12.9 cm field of view to observe three mice simultaneously, excitation filter = 570 nm, emission filter = 620 nm, f-stop 1, pixel binning 8 and 0.5 s exposure time. Images were analyzed using Living Imaging software and the Region of Interest (ROI) function. Following FLI, BLI was performed. *D*-luciferin (80 µL of 40 mg/mL sodium salt, Gold Biotechnology, St Louis, MO) was administered subcutaneously in the neck region and image acquisition started immediately with a series of images over 40 mins, as described previously [34]. BLI used the same field of view, f-stop and binning as FLI, but 1 s exposure time. Mice were then injected i.p. with vehicle [150 µL DMSO/Tween-80/saline (1∶3∶12)] and allowed to wake up. FLI and BLI (with new injections of luciferin) were repeated after 2, 4, 6, and 24 hours. The 24 hour time point served as baseline for study of therapeutic response. At this stage the three additional mice were also imaged for baseline. Then each mouse received i.p. injection of BPR0L075 (150 µL equivalent to a dose of 50 mg/kg). BLI and FLI were again repeated after 2, 4, 6, and 24 hours. The dynamic light intensity-time curves were evaluated based on total photon flux (photons/sec) values. On at least one occasion mice were examined in BLI mode prior to injection of additional luciferin to evaluate any residual signal. For comparison, MDA-MB-231-luc cells (1×10^6^) were implanted into the mammary fat pads of nude mice and BLI was performed in mice treated with vehicle or BPR0L075 in a similar manner.

### Immunohistochemistry

Histological sections of control and BPR0L075-treated (after i.p. injection of a single dose of 50 mg/kg BPR0L075) human MCF7-luc-GFP-mCherry and MDA-MB-231-luc tumors were stained with H&E. The mCherry expression was confirmed by direct fluorescence microscopy. Luciferase expression was evaluated by staining with monoclonal mouse anti-luciferase mAb (1∶150; Serotec, UK) and Cy3-conjugated goat anti-mouse secondary antibody (Jackson Laboratories, West Grove, PA). To confirm that BPR0L075 induced the disruption of tumor vascular perfusion, tumors were grown in additional mice. At each time point after BPR0L075 administration, 100 µL of the blue fluorescent dye Hoechst 33342 (10 mg/kg, Molecular Probes, Eugene, OR) was injected into the tail vein of anesthetized mice and the tumors were excised 1 min later. Tumor tissue was snap-frozen in optimal cutting temperature compound (OCT; Sakura Finetek, Torrance, CA) and stored at −80°C. A series of 8-µm cryosections was cut and fixed in 4% paraformaldehyde for 15 min at room temperature. The tissue was then washed three times in PBS for 5 minutes each cycle. After blocking with normal goat serum for 3 hours, the slides were incubated overnight at 4°C with primary rat anti-mouse endothelial marker CD31 antibody (1∶1000, BD Pharmingen, San Diego, CA). Slides were rinsed three times at 5-minute intervals with PBS and incubated with Alexa Fluor 488 goat anti-rat antibody (1∶1000, Molecular Probes, Eugene, OR) for two hours in the dark. Upon completion of secondary antibody binding, slides were washed five times using PBS for 5 minutes each cycle. The slices were mounted with fluorescent mounting medium (Dako North America, Carpinteria, CA), and imaged using an LSM 510 Meta confocal microscope (Carl Zeiss Microscopy, Germany). Tissue perfusion was revealed by cells stained with blue Hoechst 33342 dye and imaged under ultraviolet light (330–380 nm). Vascular endothelium was visualized by anti-CD31 green fluorescence of the same field. NIH-Image J software (NIH, Bethesda, MD) was used to quantify the degree of staining on images by measuring the pixels and the data were normalized to that of corresponding control mice. The extent of necrosis was also estimated using NIH-Image J software by assessing H& E stained tumor sections.

### Pharmacokinetics

CD-1 mice were injected with BPR0L075 [50 mg/kg in DMSO/Tween-80/saline (1∶3∶12)] i.p. (n = 3–4 for each time point). Time dependent biodistribution studies were carried out by sacrificing mice at 2 min, 5 min, 10 min, 30 min, 1 h, 3 h, 6 h, 9 h, and 24 h post injection. At the time of euthanasia, blood samples (∼200 µL) were collected by cardiac puncture. Plasma was obtained by centrifugation at 4°C (5000×g; 5 min) and 50 µL of plasma were spiked with 5 µL of internal standard [1-(1H-indol-3-ylcarbonyl)-1*H*-imidazole] and extracted with 135 µL of acetonitrile. The LC-MS/MS analysis of BPR0L075 was performed on a Varian 1200 LC system coupled to a triple quadrupole mass spectrometer through an electrospray ionization source in the positive ion mode (Varian, Palo Alto, CA, USA). The chromatographic separation was performed on a Varian C18 column (150 mm×2.0 mm, 5 µm particle size) with a MetaGuard column at ambient temperature. The mobile phase consisted of water (0.5% formic acid) and acetonitrile (0.5% formic acid) at 30∶70 (v/v) with a flow rate of 0.2 mL/min. The injection volume was 20 µL. Nitrogen was used as the nebulizing gas and argon gas was the collision gas. The electrospray capillary voltage was 5 kV. The API housing and drying gas temperatures were 50 and 350°C, respectively. Selected reaction monitoring (SRM) of the precursor-product ion transitions m/z 342→195 for BPR0L075, and 212→144 for internal standard were used for quantification.

### *In vitro* cytotoxicity assay

All cytotoxicity assays were performed in 96-well plates using a sulforhodamine B (SRB) colorimetric assay. Cells were plated at 2500–5000 cells/well in 100 µL of complete medium. After overnight incubation to allow attachment, a stock solution of BPR0L075 (in ethanol) was diluted to the desired concentrations in 100 µL of medium and added to cells in replicates of 16 wells per condition. Control wells received ethanol in complete medium equivalent to the maximum final ethanol concentration of drug-treated wells. After incubation for various times, cell monolayers were fixed with 10% (wt/vol) trichloroacetic acid and stained with SRB solution for 30 min, after which excess dye was removed by washing repeatedly with 1% (vol/vol) acetic acid solution. The protein-bound dye was dissolved in 10 mM Tris base solution for optical density determination at 570 nm using a microplate reader (BioTek Instruments, Inc., Winooski, VT). The concentration of drug cytotoxic and/or growth inhibitory for 50% of cells (IC_50_) was calculated using CalcuSyn 2.0 software (Biosoft, Cambridge, UK).

### Capillary disruption assays

96-well plates were coated with ice-cold Matrigel (50 µL/well), which was allowed to polymerize at room temperature for about 30 min, after which 100 µL of a suspension of HUVEC (10,000 cells/well) was seeded onto the Matrigel and cultured in medium for 12 hours to establish the capillary network. Then the ethanol vehicle or 10 nM BPR0L075 was added and the cells were monitored using an inverted phase-contrast light microscope (Olympus, Japan). Digital photographs were taken of the central area of each well at a magnification of ×200. A rat aortic ring sprouting *ex vivo* assay was performed, as described previously with modifications [35], [36]. Briefly, the thoracic aorta from a Sprague Dawley rat was excised and cross-sectioned into 1 mm-thick rings using a fine blade after the removal of all associated tissue. The rings were rinsed several times with endothelial growth medium and were embedded in the center of the 24-well plate that was precoated with 150 µL of Matrigel. An additional 200 µL of Matrigel∶RPMI (1∶1) medium containing 20 ng/mL recombinant human VEGF (Sigma A7259) was added to each well to submerge the aortic rings, and the rings were incubated at 37°C for 48 hours to establish microvascular-like sprouts. Then ethanol vehicle or 10 nM of BPR0L075 was added to the rings for 24 hours and photographs were taken by light microscope.

### Statistical analysis

Experimental values are expressed as mean ± standard deviation (SD) unless stated otherwise. Differences between means of the experimental groups and the control groups were tested using unpaired two-sided Student's *t*-test. Differences with *P*<0.05 were considered significant. BLI and FLI signals were compared using Analysis of Variance (ANOVA) on the basis of Fisher's Protected Least Significant Difference (PLSD; Statview, SAS Institute, Inc., Cary, NC) to compare mean changes at multiple time points following interventions.

## Supporting Information

Figure S1 MDA-MB-231-luc breast tumor response to administration of BPR0L075 observed by BLI. (A) BLI of control mouse at various time points after receiving vehicle; (B) Dynamic BLI showing evolution of signal intensity over 35 minutes after administration of luciferin to control mouse; respective curves show baseline (blue diamonds) together with 2 (red squares), 4 (green triangles), and 24 (purple crosses) hours after administration of vehicle; (C) Representative BLI of the mouse with respect to BPR0L075 (50 mg/kg i.p.); (D) Dynamic BLI with respect to treatment with BPR0L075: baseline (blue diamonds); 2 hours after drug (red squares), 4 hours after drug (green triangles), and 24 hours after drug (purple crosses).
(TIF)

Figure S2 Acute response of MDA-MB-231-luc breast tumors to administration of BPR0L075 observed by BLI. Normalized maximum BLI signal observed post luciferin injection at each time point sequentially for individual mice over 24 hours with respect to vehicle (n = 3, dashed lines) or drug (n = 3; solid lines). Control mice showed highly reproducible signal, though with significant increase after 24 hours, whereas drug treated mice showed significantly decreased signal at the 2 hour time point often followed by progressive recovery over 24 hours.
(TIF)

Figure S3 Histological confirmation of vascular shutdown in MDA-MB-231-luc mammary fat pad tumors. (A) Tumor sections from control tumors showing vascular extent based on anti-CD31 (green) and perfusion marker Hoechst 33342 (blue); and (B) corresponding tumor sections from tumors in mice treated with BPR0L075 (50 mg/kg i.p.) (original magnification ×400). H&E stained sections are shown for the 24 h time points at the right with both magnified fields (original magnification ×200) and whole mount revealing 17 vs. 61% necrotric fraction for control versus treated tumors.
(TIF)
