# Supplementary figures and images for: Tubulin-Destabilizing Agent BPR0L075 Induces Vascular-Disruption in Human Breast Cancer Mammary Fat Pad Xenografts
Source: PLoS One. 2012 Aug 24;7(8):e43314. doi: 10.1371/journal.pone.0043314 (PMC3427339; doi:10.1371/journal.pone.0043314)

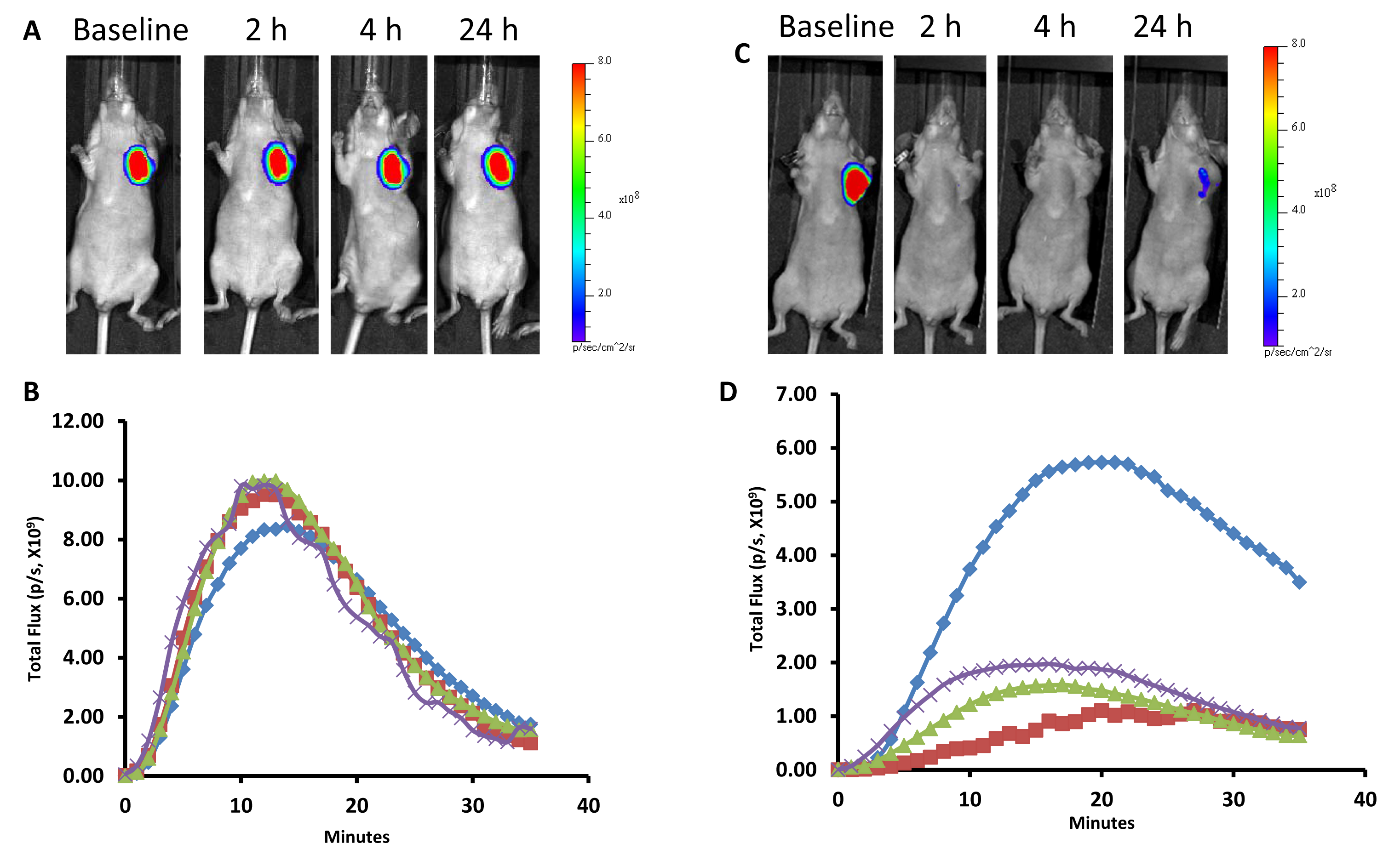

Supplement: Figure S1 — MDA-MB-231-luc breast tumor response to administration of BPR0L075 observed by BLI. (A) BLI of control mouse at various time points after receiving vehicle; (B) Dynamic BLI showing evolution of signal intensity over 35 minutes after administration of luciferin to control mouse; respective curves show baseline (blue diamonds) together with 2 (red squares), 4 (green triangles), and 24 (purple crosses) hours after administration of vehicle; (C) Representative BLI of the mouse with respect to BPR0L075 (50 mg/kg i.p.); (D) Dynamic BLI with respect to treatment with BPR0L075: baseline (blue diamonds); 2 hours after drug (red squares), 4 hours after drug (green triangles), and 24 hours after drug (purple crosses). (TIF) [file pone.0043314.s001.tif]

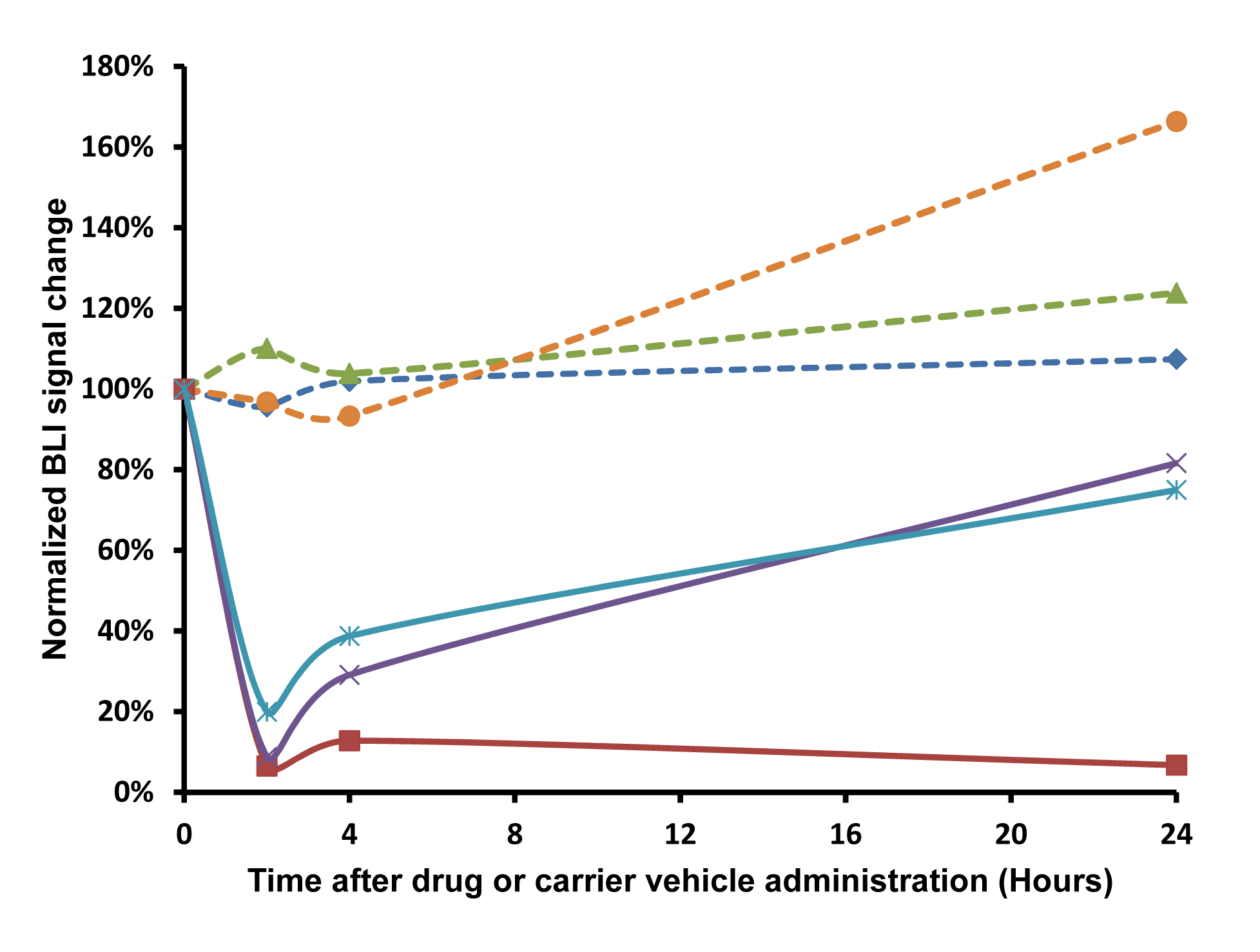

Supplement: Figure S2 — Acute response of MDA-MB-231-luc breast tumors to administration of BPR0L075 observed by BLI. Normalized maximum BLI signal observed post luciferin injection at each time point sequentially for individual mice over 24 hours with respect to vehicle (n = 3, dashed lines) or drug (n = 3; solid lines). Control mice showed highly reproducible signal, though with significant increase after 24 hours, whereas drug treated mice showed significantly decreased signal at the 2 hour time point often followed by progressive recovery over 24 hours. (TIF) [file pone.0043314.s002.tif]

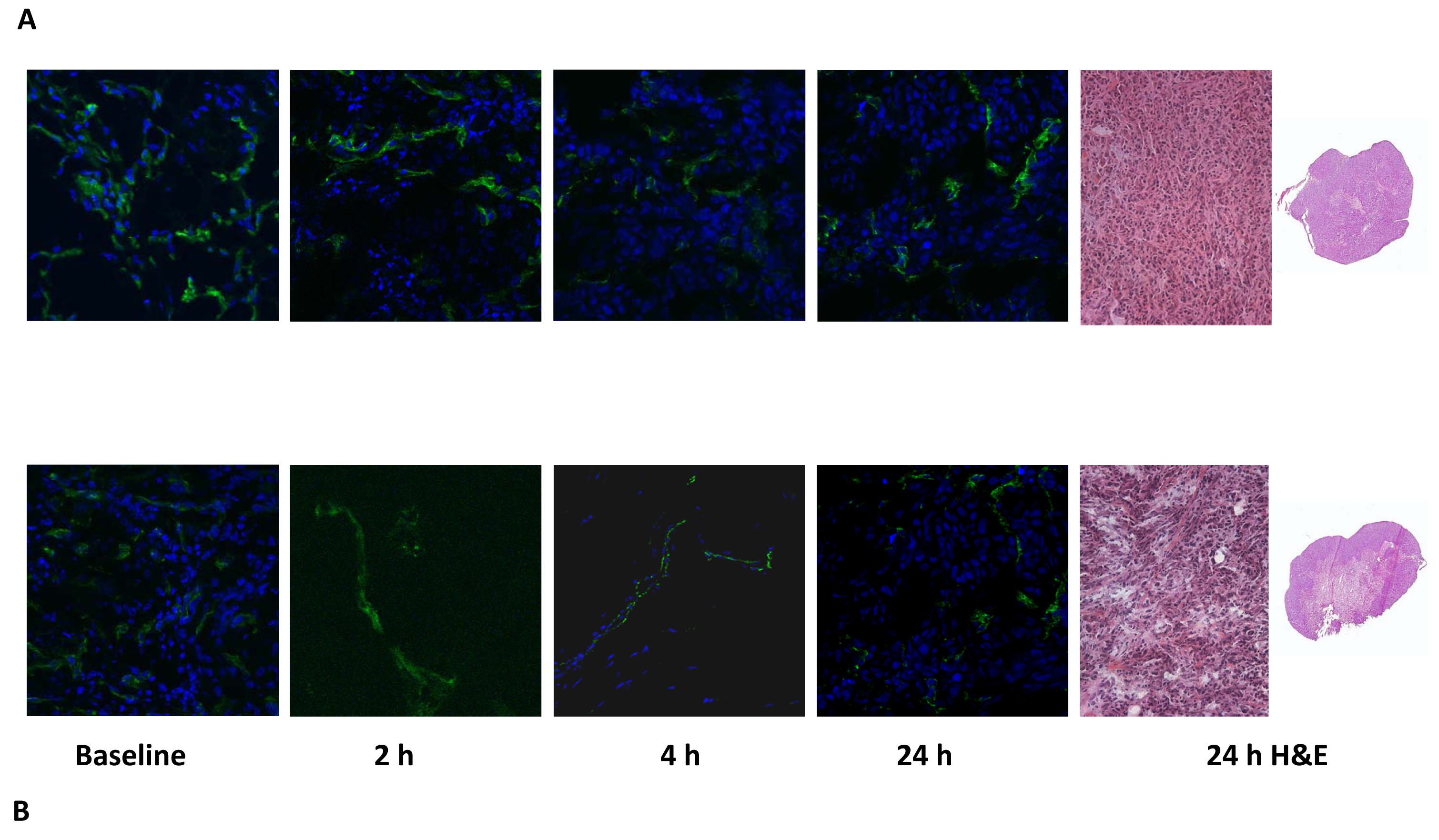

Supplement: Figure S3 — Histological confirmation of vascular shutdown in MDA-MB-231-luc mammary fat pad tumors. (A) Tumor sections from control tumors showing vascular extent based on anti-CD31 (green) and perfusion marker Hoechst 33342 (blue); and (B) corresponding tumor sections from tumors in mice treated with BPR0L075 (50 mg/kg i.p.) (original magnification ×400). H&E stained sections are shown for the 24 h time points at the right with both magnified fields (original magnification ×200) and whole mount revealing 17 vs. 61% necrotric fraction for control versus treated tumors. (TIF) [file pone.0043314.s003.tif]
